# Case report of unexpected gastrointestinal involvement in type 1 Gaucher disease: comparison of eliglustat tartrate treatment and enzyme replacement therapy

**DOI:** 10.1186/s12881-017-0403-x

**Published:** 2017-05-15

**Authors:** Yoo-Mi Kim, Dong Hoon Shin, Su Bum Park, Chong Kun Cheon, Han-Wook Yoo

**Affiliations:** 1 Department of Pediatrics, College of Medicine, Pusan National University Children’s Hospital, Yangsan, Korea; 2 Department of Pathology, College of Medicine, Pusan National University Yangsan Hospital, Yangsan, Korea; 3 Department of Internal Medicine, College of Medicine, Pusan National University Yangsan Hospital, Yangsan, Korea; 4 Medical Genetics Center, Asan Medical Center, University of Ulsan College of Medicine, Seoul, Korea; 5 Department of Pediatrics, Asan Medical Center Children’s Hospital, University of Ulsan College of Medicine, 88, Olympic-ro 43-gil, Songpa-gu, Seoul 138-736 Korea; 6 Department of Pediatrics, Pusan National University Children’s Hospital, Pusan National University School of Medicine, Geumo-ro, Yangsan-si, Gyeongnam 602-739 Korea

**Keywords:** Gaucher disease, Duodenal involvement, Enzyme replacement therapy, Eliglustat tartrate

## Abstract

**Background:**

Gastrointestinal involvement in Gaucher disease is very rare, and appears to be unresponsive to enzyme replacement therapy (ERT).

**Case presentation:**

Here, we describe identical twin, splenectomized, non-neuronopathic Gaucher patients on long-term ERT for 9 years, who complained of epigastric discomfort due to Gaucher cell infiltration of the gastroduodenal mucosa. Rare compound heterozygous mutations (p.Arg48Trp and p.Arg257Gln) of the *GBA* gene were found in both. Improvement in the gastroduodenal infiltration and reduced chitotriosidase levels were observed in one who switched to eliglustat tartrate for 1 year, whereas the other one who maintained ERT showed no improvement of chitotriosidase level and persistent duodenal lesions.

**Conclusion:**

This shows that eliglustat might be an effective treatment for Gaucher disease patients having lesions resistant to ERT.

## Background

Gaucher disease (GD) is an autosomal recessive lysosomal storage disorder resulting from a deleterious mutation in the *GBA* gene encoding β-glucosidase [1]. A deficiency in this enzyme leads to the accumulation of glycosphingolipids in the reticuloendothelial system (RES), resulting in anemia, thrombocytopenia, hepatosplenomegaly, skeletal deformation, and neurological impairment [2, 3]. Although enzyme replacement therapy (ERT) is an effective treatment for organomegaly, anemia, thrombocytopenia, and bone crisis, there are still unmet GD needs and co-morbidities despite ERT [4–6]. Moreover, unusual organ involvement, such as the lungs, mesenteric lymph nodes, or gastrointestinal (GI) tract, is difficult to treat with ERT. Herein, we report unusual GI mucosal involvement in identical twin Korean siblings with non-neuronopathic GD during long-term ERT, and clinical improvement 1 year after the administration of eliglustat tartrate.

## Case report

### Subject 1

A 35-year-old man, the elder of identical twins, was diagnosed with GD at the age of 9 years old via a bone marrow biopsy, when he was brought to medical attention because of hepatomegaly and recurrent osteomyelitis. The patient underwent a splenectomy at the age of five because of progressive massive splenomegaly and thrombocytopenia. He began ERT treatment (imiglucerase, Cerezyme®) at age 26, when this treatment became available. The patient received ERT at a dosage of 60 units/kg every other week, and was referred to our hospital; however, he transiently received one-half of a dose of ERT during the global enzyme shortage for 2 years. He did not show any neurological signs, including impaired initiation of saccades, horizontal gaze palsy, seizure, or cognitive decline, but his white blood cell glucocerebrosidase activity was 1.1 nmol/h/mg (normal 10.3–41.8). He was subjected to direct sequencing of the *GBA* gene, which showed the compound heterozygous mutations c.259C > T (p.R48W) and c.5118G > A (p.R257Q). The laboratory findings, including biomarkers, showed levels with the normal ranges: hemoglobin 15.5–16 mg/dl (normal 14–17), platelet count 190,000–200,000/μl (normal 140,000–400,000), acid phosphatase 2.5–5.2 U/l (normal 0–6.6), and angiotensin-converting enzyme (ACE) 47–58 U/l (normal 20–70). The chitotriosidase level was 958.6 nmol/h/ml (normal 4–76) during his visit to our hospital. His previous result could not be obtained.

**Fig. 1:**
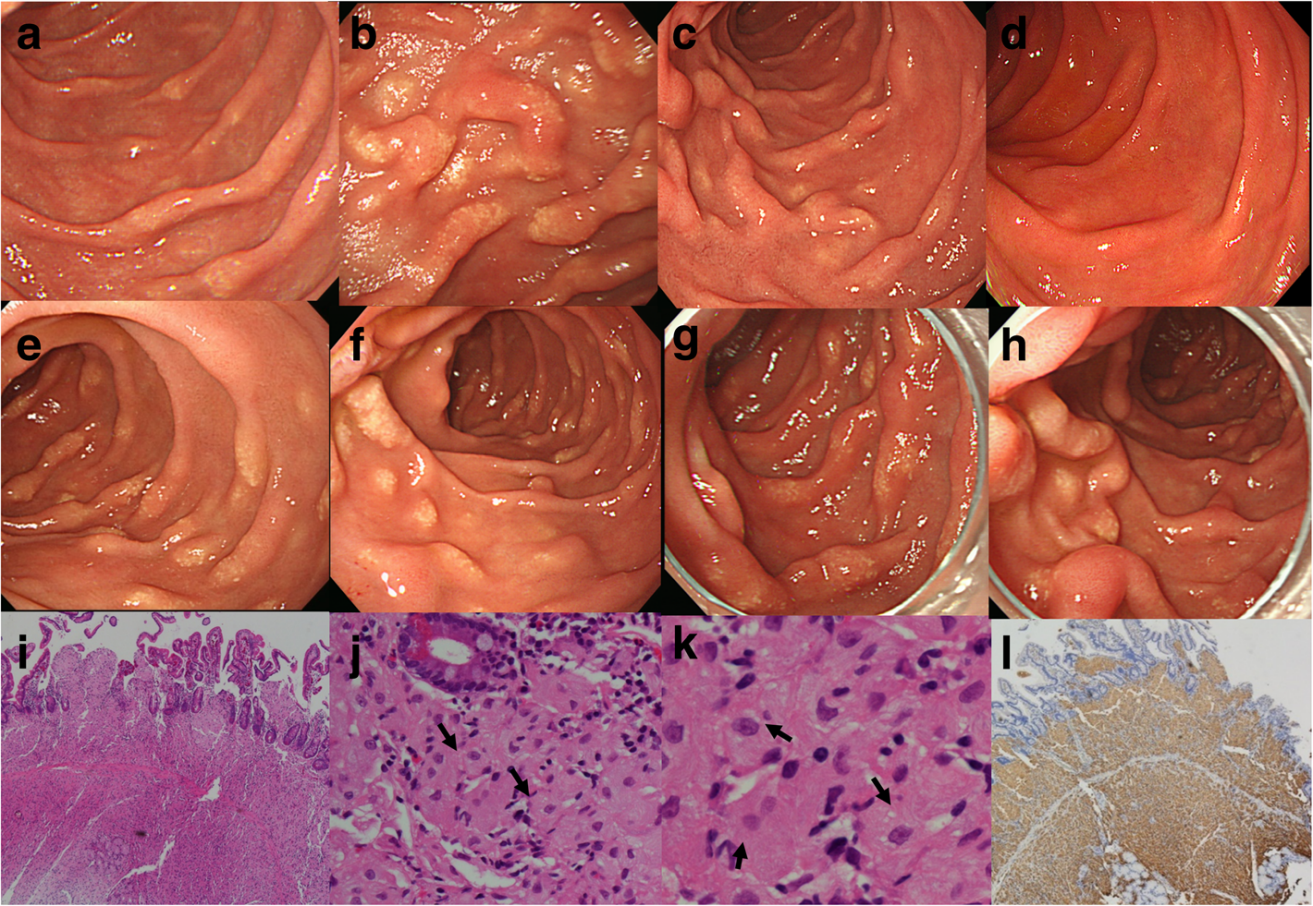
Gross and pathological findings of the duodenal mucosa in Cases 1 and 2. Gaucher cell infiltration presenting with multiple yellowish nodular lesions in the upper gastrointestinal (GI) endoscopy at baseline (**a**), persistent lesions 6 months after a high dose of enzyme (**b**), slightly improving multiple nodular lesions at 6 months (**c**), and grossly improving at 12 months (**d**) after eliglustat tartrate administration in Case 1. Persistent multiple nodular lesions in Case 2 at baseline (**e**, **f**), and 12 months (**g**, **h**) after ERT. Gaucher cell infiltration in the duodenal mucosa at baseline (H&E, ×40, ×200, ×400) (**i**, **j**, **k**). Gaucher cells are strongly positivity for CD68, which is a special stain for macrophages and Gaucher cells (CD68, ×40) (**l**)

Although this patient had been on ERT for more than 9 years, the skeletal involvement progressed to avascular necrosis of the hip joint; however, he complained of epigastric discomfort and poor weight gain. Over the past 2 years, his body weight slowly decreased from 48 kg (body mass index [BMI], 18.7 kg/m^2^) to 42 kg (BMI, 16.4 kg/m^2^), and he was unable to consume pills or food easily because of epigastric discomfort. His pain was aggravated by spicy food. Despite the weight loss, his total protein level was 7.1–7.6 gm/mL (normal 6.6–8.3). An upper GI endoscopy for dyspepsia was performed by a local hospital 2 years previously, revealing mild gastritis. Due to the intractable epigastric discomfort, the upper GI endoscopy was followed up, and showed multiple nodular yellowish lesions on the duodenum (Fig. 1a). The pathological findings of the biopsy specimen revealed that the nodular lesions consisted of Gaucher cell infiltration, and stained positively with CD68 (Fig. 1i–l).

The immunoglobulin G (IgG) antibody against imiglucerase was negative, and we gradually increased the dose of imiglucerase from 60 units/kg to 100 units/kg every other week. Despite the high dose of ERT for 6 months, a mild reduction in the chitotriosidase level (from 752.5 to 695.3 nmol/h/ml) was observed, but the upper GI endoscopy showed that the duodenal lesions remained unchanged (Fig. 1b). Additional colonoscopy and whole-body magnetic resonance imaging (MRI) did not show any new lesions, including mesenteric lymph node involvement. After *CYP2D6* genotyping, which revealed an extensive metabolizer, was conducted, the ERT was switched to eliglustat tartrate (supported by Sanofi Genzyme through the named patient program) at 100 mg (84 mg for eliglustat alone) two times daily. Every 6 months, an upper GI endoscopy and blood testing for biomarkers were conducted during substrate reduction therapy (Fig. 1c and d). After 1 year of eliglustat tartrate treatment, the upper GI endoscopy showed gross improvement in the duodenal lesions (Fig. 1d), a remarkable reduction in the chitotriosidase levels (from 717.5 to 257.5 nmol/h/ml), and an increased body weight (to 50 kg, BMI 19.5 kg/m^2^). No adverse events were observed from the eliglustat tartrate treatment.

### Subject 2

A 35-year-old man, the younger of identical twins, was diagnosed with GD at the age of 9 years old via a bone marrow biopsy, when he presented with hepatomegaly and recurrent osteomyelitis. At the age of 5 years old, he underwent a splenectomy for massive splenomegaly. This patient had been receiving ERT with 60 units/kg of imiglucerase every 2 weeks since he was 26 years old, and showed no neurological symptoms. He also experienced a dose reduction of ERT during the shortage period. The leukocyte glucocerebrosidase activity in this patient was 1.9 nmol/h/mg (normal 10.3–41.8). He lost 6 kg in the past 2 years (body weight: 44 kg), and his BMI decreased to 17.6 kg/m^2^, although he did not complain of severe dyspepsia like his older brother did. His total protein was 7.0–7.5 gm/dl (normal 6.6–8.3). The chitotriosidase level during his visit to our hospital was 1335.7 nmol/h/ml. Other biomarkers, including ACE and acid phosphatase, were in normal.

An upper GI endoscopy was performed, and nodular yellowish lesions on the duodenal mucosa indicated Gaucher cell infiltration. The IgG antibody against imiglucerase was also negative in this patient. As subject 2 was concerned about adverse events and the effectiveness of a high dose of ERT or eliglustat, he did not want to increase the dosage of ERT or switch to eliglustat tartrate as his older brother had. Thus, the 60 units/kg dose of imiglucerase every 2 weeks was maintained. This subject’s chitotriosidase level decreased from 1038.3 to 950.4 nmol/h/ml during same first 6 months of ERT, which was higher in the older brother, but the latest chitotriosidase level was still high (815.1 nmol/h/ml) when compared to the chitotriosidase level of the older brother treated for 1 year with eliglustat tartrate. Although his weight increased to 47 kg over 1 year, the recent upper GI endoscopy demonstrated persistent duodenal lesions in spite of the regular ERT (Fig. 1e–g). The colonoscopy and whole-body MRI did not indicate any additional lesions.

## Discussion

ERT has been proven safe and effective for visceromegaly, anemia, thrombocytopenia, and skeletal symptoms related to GD [1, 3–6], and it reduces the blood levels of the acid phosphatase, ACE, and chitotriosidase biomarkers [7]. However, several factors may have caused the unusual manifestations seen in these twin brothers, despite long-term ERT. First, the splenectomies might have aggravated the bone lesions and unusual organ involvement. In the present cases, the splenectomy was performed at 5 years old, and recurrent osteomyelitis was observed throughout childhood. Since the inception of ERT, a splenectomy has been contraindicated in cases of GD [1, 3, 4], because it increases the risk for co-morbidities via systemic infection, malignancy, cholelithiasis, pulmonary hypertension, or hepatopulmonary syndrome [2, 5–8]. In addition, it can accelerate neurological debilitation and aggravate bone disease, such as avascular necrosis of the femur neck [6, 8]. As the spleen may be a main reservoir for Gaucher cells in patients with GD, a splenectomy may aggravate the Gaucher cells to move to other organs, such as the lungs, lymphoid system, liver, GI tract, or bone marrow.

Second, the remarkable delay between the diagnosis and initiation of ERT was a critical factor, causing the constellation of severe clinical features. The third factor was that our twin brothers had unique mutations in the *GBA* gene, p.R48W and p.R257Q, which are rare. Although regarded as a mild mutation, p.R48W has been reported in non-neuronopathic GD manifesting bone disease, whereas p.R257Q has previously been reported in both acute neuronopathic and non-neuronopathic GD. Interestingly, p.R257Q was reported in a Type 2 Gaucher patient, and manifested with the infiltration of Gaucher cells throughout the body, including the brain, liver, lung, and GI tract [9]. Fourth, it seems that long-term ERT modifies the natural history of GD. For example, ERT-resistant mesenteric lymphadenopathies serve as sanctuary sites against ERT [10]. The mucosa of the GI tract may be resistant to ERT because of the relative scarcity of the mannose receptor [11]. Since the antibodies against imiglucerase were negative in our cases, the neutralizing antibody did not hamper clinical efficacy.

Since there are few reports on GI tract infiltration in GD (Table 1) [9, 12–15], no consensus guidelines on the treatment of these unusual cases have been suggested, and there are limitations for alternative treatments. We first tried to increase the dose from 60 units/kg to 100 units/kg in Case 1; however, this appeared to have little effect on reducing the Gaucher cell burden of the GI mucosa. Considering the fact that oral medication might reach and penetrate the GI mucosa better than imiglucerase, substrate reduction therapy (SRT) could have been an alternative treatment in the present cases. Miglustat was first introduced as an SRT, and showed an effectiveness similar to that of imiglucerase [16]. However, it had an adverse effect on the GI tract, including diarrhea, nausea, and vomiting, and limited indications for Gaucher disease when a patient was unable to use ERT. Eliglustat tartrate (Cerdelga®) was approved by the US Food and Drug Administration in 2014. It is an active specific inhibitor of glucosylceramide synthase with limited toxicity, a good outcome, and stability in non-neuronopathic GD, even in patients switching from ERT [17–19]. We found that switching to eliglustat tartrate to reduce the mucosal involvement of the Gaucher cells was effective, and no adverse events were observed.

**Table 1:** Clinical characteristics and genotype of patients with Gaucher disease and gastrointestinal mucosal involvement

	Case 1	Case 2	Case 3	Case 4	Case 5	Case 6	Case 7
Age at onset	33 years	33 years	fetal period	fetal period	11 months	10 years	39 years
Type	non-neuronopathic	non-neuronopathic	acute neuronopathic	acute neuronopathic	acute neuronopathic	non-neuronopathic	non-neuronopathic
Involvement sites	duodenal mucosa	duodenal mucosa	stomach, small intestine	gastrointestinal (GI) tract	gastric mucosa	colon	GI tract (from stomach to rectum)
Symptoms	severe dyspepsia, weight loss	mild dyspepsia, weight loss	hydrops fetalis	colloid baby	massive GI bleeding	hematochezia, cured	GI bleeding
Treatment	SRT (eliglustat tartrate)	ERT	none	none	high dose of ERT, PPI	endoscopic sclerotherapy, polypectomy	none
Outcome	improving	persistent	expired	expired	Expired	cured	expired
*GBA* gene	p.R48W, p.R257Q		p.R257Q, p. P171fsX21	p.R120W, p.S196P	Homozygous for p.L483P	NA	NA
Age at splenectomy	5 years	5 years	ND	ND	ND	4 years	ND
Reference	present cases		[9]	[12]	[13]	[14]	[15]

*NA* not available, *SRT* substrate reduction therapy, *ERT* enzyme replacement therapy, *PPI* proton pump inhibitor, *ND* not done

In summary, our report has clearly described the efficacy of oral SRT in one identical twin with GD. Both of these twins showed unusually persistent GI mucosal Gaucher cell infiltration, despite long-term ERT. Eliglustat tartrate may not only be a new alternative treatment for ERT, but also an effective therapy for patients especially resistant to ERT.

## Abbreviations

ACE: Angiotensin-converting enzyme
BMI: Body mass index
ERT: Enzyme replacement therapy
GBA: β-glucosidase
GD: Gaucher disease
SRT: Substrate reduction therapy
